# Molecular Characterization of the Tumor Suppressor Candidate 5 Gene: Regulation by PPAR*γ* and Identification of TUSC5 Coding Variants in Lean and Obese Humans

**DOI:** 10.1155/2009/867678

**Published:** 2010-03-01

**Authors:** Trina A. Knotts, Hyun Woo Lee, Jae Bum Kim, Pieter J. Oort, Ruth McPherson, Robert Dent, Keisuke Tachibana, Takefumi Doi, Songtao Yu, Janardan K. Reddy, Kenji Uno, Hideki Katagiri, Magdalena Pasarica, Steven R. Smith, Dorothy D. Sears, Michel Grino, Sean H. Adams

**Affiliations:** ^1^Obesity & Metabolism Research Unit, USDA-Agricultural Research Service Western Human Nutrition Research Center, Davis, CA 95616, USA; ^2^Department of Biological Sciences, Department of Biophysics and Chemical Biology, Seoul National University, Seoul 151-742, South Korea; ^3^Division of Cardiology, University of Ottawa Heart Institute, Ottawa, Canada K1H 8L1; ^4^Graduate Schools of Pharmaceutical Sciences, Osaka University, Osaka 565-0871, Japan; ^5^Department of Pathology, The Feinberg School of Medicine, Northwestern University, Chicago, IL 60208, USA; ^6^Division of Advanced Therapeutics for Metabolic Diseases, Tohoku University Graduate School of Medicine, Sendai 980-8576, Japan; ^7^Pennington Biomedical Research Center, Baton Rouge, LA 70808, USA; ^8^Department of Medicine, University of California, San Diego, La Jolla, CA 92093, USA; ^9^Institute National de la Santé et de la Recherche Médicale (INSERM) UMR 626, 13385 Marseille and Faculté de Médecine, Université de la Méditerranée, Marseille, France; ^10^Department of Nutrition, University of California, Davis 95616, USA

## Abstract

Tumor suppressor candidate 5 (TUSC5) is a gene expressed abundantly in white adipose tissue (WAT), brown adipose tissue (BAT), and peripheral afferent neurons. Strong adipocyte expression and increased expression following peroxisome proliferator activated receptor *γ* (PPAR*γ*) agonist treatment of 3T3-L1 adipocytes suggested a role for Tusc5 in fat cell proliferation and/or metabolism. However, the regulation of Tusc5 in WAT and its potential association with obesity phenotypes remain unclear. We tested the hypothesis that the TUSC5 gene is a bona fide PPAR*γ* target and evaluated whether its WAT expression or single-nucleotide polymorphisms (SNPs) in the TUSC5 coding region are associated with human obesity. Induction of Tusc5 mRNA levels in 3T3-L1 adipocytes by troglitazone and GW1929 followed a dose-response consistent with these agents' binding affinities for PPAR*γ*. Chromatin immunoprecipitation (ChIP) experiments confirmed that PPAR*γ* protein binds a ∼ −1.1 kb promotor sequence of murine TUSC5 transiently during 3T3-L1 adipogenesis, concurrent with histone H3 acetylation. No change in Tusc5 mRNA or protein levels was evident in type 2 diabetic patients treated with pioglitazone. Tusc5 expression was not induced appreciably in liver preparations overexpressing PPARs, suggesting that tissue-specific factors regulate PPAR*γ* responsiveness of the TUSC5 gene. Finally, we observed no differences in Tusc5 WAT expression or prevalence of coding region SNPs in lean versus obese human subjects. These studies firmly establish the murine TUSC5 gene locus as a PPAR*γ* target, but the significance of Tusc5 in obesity phenotypes or in the pharmacologic actions of PPAR*γ* agonists in humans remains equivocal.

## 1. Introduction

Tumor suppressor candidate 5 (TUSC5, also known as LOST1 or BEC-1) was originally identified as a gene locus disrupted in some lung cancers and, therefore, hypothesized to participate in attenuation of cancer cell proliferation [1]. Consistent with this postulate, Tusc5 protein contains a CD225 interferon-induced transmembrane protein domain [2], found in the archetypal interferon-responsive 9–27 protein that is implicated in antiproliferative actions of interferons [3]. It is now firmly established that Tusc5 expression is remarkably tissue specific in rodents and humans, with robust expression in mature white and brown adipocytes [2, 4, 5] and in peripheral afferent neurons [2]. This unique expression pattern suggests an important function for Tusc5 in both adipose tissue and the peripheral nervous system.

With respect to fat tissue, we proposed a working hypothesis in which Tusc5 participates in pathways modulating adipocyte proliferation or promoting cell cycle growth arrest/fat cell maturation in response to environmental or central nervous system cues [2]. This model was based largely on the putative tumor suppressor features of Tusc5 (see above), coincident expression of Tusc5 with adipocyte markers that increase as 3T3-L1 adipocytes exit the mitotic clonal expansion phase to terminally differentiate and mature [2], and the repression of Tusc5 expression in the “proliferative” brown adipose tissue (BAT) of cold exposed rodents ([4]; also see [2]). According to this model, Tusc5 has a “governor” role that would be dampened when adipocyte growth cues are triggered (i.e., by cold or obesity). However, the exact physiological function of Tusc5 has remained elusive.

Despite recent advancements in the characterization of Tusc5 biology in fat cells, its gene regulators and potential links to obesity phenotypes remain to be clarified. Identifying factors that influence Tusc5 gene expression and gaining a better understanding of associations between the TUSC5 gene sequence, expression patterns, and obesity will enable a deeper understanding of its physiological function. Previously, we reported that Tusc5 mRNA and protein abundance increase following short-term (∼1 day) or 1-week treatment of 3T3-L1 adipocytes with a high concentration of the peroxisome proliferator activated receptor *γ* (PPAR*γ*) agonist GW1929 [2], supporting the hypothesis that TUSC5 is a bona fide PPAR*γ* target gene. If confirmed, this would bolster the view that Tusc5 is a metabolically-relevant factor, since PPAR*γ* target genes typically participate in adipocyte function and other important metabolic processes. To address this question further, we searched for potential PPAR*γ*-response elements (DR1 sites) in the murine TUSC5 promoter, performed chromatin immunoprecipitation (ChIP) studies to determine whether PPAR*γ* interacts with these sites during 3T3-L1 adipogenesis, and assessed whether these interactions take place concurrent with changes in chromosomal histone acetylation. To gain insight into the clinical relevance of PPAR*γ*-TUSC5 interactions, Tusc5 mRNA levels were compared in archived human WAT biopsy samples before and after treatment with the PPAR*γ* agonist pioglitazone or rosiglitazone. In a complementary line of research, we examined relationships between obesity and Tusc5, by comparing TUSC5 gene coding variants and its WAT mRNA expression in obese and nonobese human subjects. We hypothesized that WAT Tusc5 transcript levels would be reduced during states characterized by white adipocyte proliferation, as in obesity, and that the frequency of sequence variation in the TUSC5 coding region would differ between lean versus obese humans. Our results support the view that murine TUSC5 is a PPAR*γ* target gene, but a relationship between Tusc5, obesity, and pharmacological actions of PPAR*γ* agonists remains equivocal.

## 2. Methods

**Materials**

Insulin, dexamethasone, 3-isobutyl-1-methylxanthine (IBMX), and GW1929 were purchased from Sigma. DMEM and Superscript III First-strand synthesis kit were purchased from Invitrogen. Tissue culture dishes and multiwell plates were from BD Falcon. Newborn calf serum was obtained from Hyclone and lot-tested fetal bovine serum (FBS) was purchased from Atlas Biologicals.

### 2.1. Experiments to Evaluate the Tusc5 Gene Locus as a PPAR*γ* Target

**3T3-L1 Adipocyte Differentiation and PPAR*γ* Agonist Dose-Response Studies**

The impact of treatment with GW1929 and troglitazone on Tusc5 mRNA abundance was tested in the murine 3T3-L1 adipocyte model. Cells were grown as described previously [2], except insulin was withdrawn after the initial two-day differentiation period and cells were grown in 6-well uncoated plates. Mature adipocytes (10–12 days postdifferentiation, media changed every 3-4 days) were grown for 18 hours in media containing the potent non-thiazolidinedione PPAR*γ* agonist GW1929 [6] or the less potent thiazolidinedione (TZD) agonist troglitazone in order to generate a dose-response relationship of Tusc5 expression (*n* = 4/per dose); controls were grown in media containing dimethyl sulfoxide vehicle (DMSO; 0.1% by vol.). The experiment was replicated twice. RNA was prepared using Trizol-based methods for cell culture samples as per manufacturer's instructions (Ambion, Austin, TX), and transcript abundances of target genes were measured as described under “Gene Expression Analyses” Section below. Parallel-treated plates were used to generate protein lysates for measurement of Tusc5 protein by Western blot, as described below.

**Chromatin Immunoprecipitation (ChIP) Assays**

ChIP assays were performed as described previously [7]. In brief, confluent preadipocytes (day 0) and differentiated 3T3-L1 adipocytes (at day 4 or 8 following addition of differentiation cocktail, which was added over days 1-2) were cross-linked in 1% formaldehyde at 37°C for 10 minutes and resuspended in 200 *μ*L of Nonidet P-40-containing buffer (5 mM PIPES, pH 8.0, 85 mM KCl, and 0.5% NP-40). Crude nuclei were isolated and lysed in 200 *μ*L lysis buffer (1% SDS, 10 mM EDTA and 50 mM Tris-HCl, pH 8.1), and lysates were sonicated and diluted 10-fold with immunoprecipitation buffer (16.7 mM Tris-HCl [pH 8.1], 167 mM NaCl, 1.2 mM EDTA, 0.01% SDS, and 1.1% Triton X-100). Lysates were immunoprecipitated with antiacetylated-histone H3 (K9) (1 *μ*g), antiacetylated-histone H4 (pan)(1 *μ*g), or antiPPAR*γ* (1 *μ*g) antibodies for 12 hours at 4°C. Immune complexes were incubated with Protein A-Sepharose CL-4B (Amersham-Biosciences) for 2 hours at 4°C. “Input” represents 10% of the total input chromatin. After successive washings, immune complexes containing DNA were eluted and the precipitated DNA was amplified by PCR. These experiments were replicated three times. Promoter primer pairs used in this study are as follows: (1) 301 bp amplicon for putative −8 kb DR-1 (5′: GTTCCACATATGTTGAACT, 3′: GAAGGAAGAAAGACAGACT); (2) 289 bp amplicon for putative −1.8 kb DR-1 (5′: CACCAAGCAAACATGCTTT, 3′: ACAACATGCACGTAAGTGC); (3) 304 bp amplicon for putative −1.1 kb DR-1 (5′: AAAGCCACCCTTCCCATAC; 3′: CCTAAAGCCACCAAGGGAA); (4) nonspecific 374 bp amplicon near the putative −8 kb DR-1 sequence (5′: AGTCTGTCTTTCTTCCTTC, 3′: TGCTACAAGAAACCTTTCA). Antibodies against acetylated-histone H3 (K9), acetylated-histone H4 (pan) were purchased from Upstate Biotechnology, and the antibody against PPAR*γ* was from Abcam.

**Tusc5 WAT Transcript Abundance Following Treatment with TZDs in Subjects with Type 2 Diabetes Mellitus (T2DM)**

In the first study, archived samples were available from a subset of volunteers involved in a study previously described [8]. Subjects with T2DM were treated with pioglitazone (PIO) (30 mg/day; *n* = 16: 5 male, 11 female) or placebo (*n* = 16: 7 male, 9 female) for 11–17 weeks. If fasting plasma glucose was >100 mg/dL or HbA1c was ≥7.0% at week 8 of the study, the dose of PIO was increased to 45 mg/d. Subcutaneous abdominal adipose tissue (WAT) biopsies were obtained by Bergstrom needle at the beginning and end of the study after an overnight fast, with local Lidocaine anesthesia, and samples were flash frozen and stored at −80°C until processed for mRNA and target gene transcript quantitation as described below. Clinical studies were approved by the Institutional Review Board of the Pennington Biomedical Research Center.

In a second line of research, archival tissue samples were from 27 type 2 diabetic subjects, participants in studies where the objectives were to generate expression profiles before and after TZD treatment. Some reports from the individual clinical studies have been published previously [9, 10]. The subjects are a subset of a larger, three-study cohort used to study expression profiles related to insulin resistance and TZD treatment [11]. The pioglitazone study included eight subjects (all male, age 48 ± 3 years, BMI 36.5 ± 2.8 kg/m^2^, fasting plasma glucose 173 ± 19 mg/dL). The rosiglitazone study included 19 subjects (6 female/13 male, age 52 ± 2 years, BMI 35.6 ± 1.4 kg/m^2^, fasting plasma glucose 183 ± 15 mg/dL). Needle biopsies of abdominal subcutaneous adipose tissue were harvested before and after 12-week treatment with pioglitazone (45 mg/day) or rosiglitazone (8 mg/day). Biopsies were flash frozen in liquid nitrogen and stored at −80°C. Oligonucleotide microarrays (Human Genome U133 Plus 2.0, Affymetrix, Inc., Santa Clara, CA) were used to generate gene expression profiles from each adipose tissue sample. RNA preparation and array analyses have been described [11]. Gene expression levels, expressed as average difference scores, were determined using Affymetrix MAS 5.0 software. The experimental protocols were approved by the Institutional Review Board for Human Subjects of the University of California, San Diego, and informed written consent was obtained from each subject. 

### 2.2. Studies of Tusc5 Gene Activation by PPAR*γ* Outside the Context of Adipocytes

**Analyses of Tusc5 Gene Expression in Livers of Mice Overexpressing PPAR*γ*1 or PPAR*γ*2 in Liver**

For examination of the effect of liver PPAR*γ*1 overexpression on expression of Tusc5, samples were used from a previously published study in which PPAR*α* knockout mice were treated with i.v. doses of adenovirus preparations containing either murine PPAR*γ*1 (*n* = 3) or LacZ control (*n* = 3) over expression contructs that drive expression primarily in liver (see [12] for details related to animal treatments and RNA isolation). Total RNA was subjected to quantitative PCR using Taqman primers and probes directed against Tusc5 or the positive control PPAR*γ* target gene adipoQ (encoding adiponectin), as described below.

For determination of the effects of liver overexpression of murine PPAR*γ*2 on hepatic expression of Tusc5, archived samples were used from a previously published experiment in which C57BL/6 mice were i.v. treated with adenovirus preparations containing either murine PPAR*γ*2 (*n* = 6) or LacZ (control, *n* = 7) overexpression constructs [13]. cDNA from total RNA (oligo −dT primed) was prepared from liver tissue, and gene expression analyses were performed as described below.

**Effects of Overexpression of PPAR isoforms in HepG2 Hepatoblastoma Cells**

These studies were conducted at Osaka University, and the tightly tetracycline (tet)-regulatable HepG2-tet-off-hPPAR (PPAR isoforms *α*, *β*/*δ*, *γ*1, *γ*2) hepatoblastoma cells used in these experiments, cell culture conditions, and RNA preparation techniques have been described elsewhere in detail [14]. Briefly, cells were cultured in Dulbecco's Modified Eagle's Medium (high glucose DMEM) containing 10% heat-inactivated FBS, 100 IU/mL penicillin, 100 *μ*g/mL streptomycin, 300 *μ*g/mL G418 antibiotic, 0.5 *μ*g/mL puromycin, and 2 *μ*g/mL Tet. For PPAR ligand treatments, cells were cultured in the medium supplemented with 10% heat-inactivated charcoal/dextran-treated FBS. To overexpress the PPAR isoforms, HepG2-tet-off-hPPAR cells (7 × 10^5^ cells/dish) were seeded in 6 cm dishes without Tet for 24 hours [14]. Cells were then treated with PPAR ligand (1 *μ*M GW7647 for PPAR*α*, 100 nM GW501516 for PPAR*δ*, or 1 *μ*M rosiglitazone for PPAR*γ*) or vehicle (0.1% DMSO) for 24 hours. Total RNA was isolated from the cells using the QuickGene RNA cultured cell HC kit (FUJIFILM) according to the manufacturer's instructions. mRNA prepared from these samples was transferred to the WHNRC for quantitative PCR analyses, as described below. RNA was quantified on a NanoDrop ND-1000 spectrophotometer and processed as described below for measurement of transcript abundances of target genes including human Tusc5 and the positive control pan-PPAR-target gene adipose differentiation related protein (ADRP or ADFP) plus the PPAR targets phosphoenolypyruvate 1 (PEPCK1/Pck1), acyl-CoA oxidase 1 (ACOX1), and carnitine palmitoyltransferase 1b (Cpt1b). There was a total of 3-sample/treatment group.

### 2.3. Studies Evaluating Tusc5 in Human Obesity

Clinical investigations were approved by the institutional review boards of each participating center and were performed according to the Declaration of Helsinki.

**Expression of Tusc5 mRNA in Obese Human WAT**

The effects of obesity and differences between adipose depots with respect to Tusc5 mRNA abundance were analyzed using archived human WAT samples from nonobese (*n* = 10) and obese (*n* = 12) subjects. Samples were derived from female volunteers undergoing surgery, as previously described [15]; samples are the same as those used for our recent characterization of another adipocyte-neuron gene, synuclein-*γ* (SNCG; [16]). Visceral adipose tissue (omental) was collected in the course of laparoscopy or laparotomy for gastroplasty or gynecological procedures, and SC fat obtained in parallel from the abdominal region. mRNA prepared from these samples was transferred to the WHNRC for quantitative PCR analyses, as described below.

**TUSC5 Sequence Variants in Lean and Obese Subjects**

Details regarding the subject population, exclusion and inclusion criteria, sequencing methods, and data analysis techniques have been previously described [17]. Genomic DNA samples obtained from a large cohort of extremely obese (*n* = 381, mean BMI 49.0 ± 8.8 kg/m^2^) and very lean (*n* = 377, mean BMI 19.4 ± 1.6 kg/m^2^) white individuals of European descent were analyzed at the U.S. Department of Energy Joint Genome Institute (JGI), in the laboratory of Dr. Len A. Pennacchio. TUSC5 (NM_172367) coding exons and their splice sites were sequenced using gene-specific primer sets, and sequence polymorphism allele frequencies were compared between the lean and obese groups. Subject recruitment was from the Ottawa community (lean subjects) or from the University of Ottawa Weight Management Clinic and the Heart Institute Lipid Clinic [17].

**Gene Expression Analyses**

RNA was prepared from 3T3-L1 adipocytes and human WAT biopsy samples using the Ribopure kit (Applied Biosystems-AM1924). RNA abundance was quantified using a NanoDrop ND-1000 Spectrophotometer (NanoDrop Technologies). cDNA was synthesized from total RNA using Superscript III reverse transcriptase (Invitrogen) followed by RNase-H treatment as per the manufacturer's instructions. Gene expression analyses by quantitative PCR utilized gene-specific Taqman primers and FAM-MGB labeled probes (Assays-on-Demand, Applied Biosystems, Inc.) and were analyzed in duplicate or triplicate for each sample using an ABI 7900HT instrument. Reactions were carried out in a 384-well format containing the following in each well: cDNA corresponding to 10 ng of original total RNA (3T3-L1 PPAR*γ* dose response studies), 2 ng (PPAR*γ*) or 10 ng (Tusc5, ARBP/36B4, AdipoQ) for murine PPAR*γ*1 and PPAR*γ*2 liver overexpression studies, 20 ng (HepG2 PPAR overexpression studies), or 5 ng (human WAT samples); cDNA was dried in each well prior to adding qPCR reagents to facilitate an 8 *μ*L/well assay. Wells also contained 1x Master Mix (ABI Universal PCR Master Mix or Taqman Gene Expression Master Mix) and 1x specific primer-probe mix. Cycle conditions were 50°C for 2 minutes, 95°C for 10 minutes, 40 cycles of 95°C for 15 s/60°C for 1 minute. Amplification cycle number (Ct) of control mRNA (eukaryotic 18S) was determined using commercial primers and probes (ABI) to correct for template loading differences, and expression values were determined relative to treatment control transcript levels using a mathematical formula as previously described [2]. Primers/probe ABI identifiers for mouse studies were Tusc5 (Mm00624784_m1), aP2/FABP4 (Mm00445880_m1), PPAR*γ* (Mm00440945_m1), and adiponectin/adipoQ (Mm00456425_m1). For human cell and tissue studies, identifiers were ADFP (Hs00605340_m1), adiponectin/AdipoQ (Hs00605917_m1), Cpt1b (Hs00992664_m1), PEPCK1/Pck1 (Hs00159918_m1), leptin (Hs00174877_m1), ACOX1 (Hs00244515_m1), and LPL (Hs00173425_m1).

**Protein Extraction and Western Blot Analysis**

Briefly, tissue samples were homogenized on ice in homogenization buffer (50 mM PIPES, 150 mM NaCl, 1.5% n-Octyl-*β*-D-glucopyranoside; 300 *μ*L/100 mg tissue) with 1X HALT protease and phosphatase inhibitors (Pierce). 3T3-L1 cells were washed once with PBS, lysed with Tusc5 homogenization buffer with inhibitors, and sonicated for 5 s. Lysates were spun at 20000 g at 4°C for 10 minutes. Protein concentrations were quantitated using the bicinchoninic acid assay (Pierce). For Tusc5, 2.5–5 *μ*g of total protein was separated on a 12% Bis-Tris SDS gel using 2-(N-morpholino)ethanesulfonic (MES) acid running buffer (Invitrogen). The proteins were transferred to polyvinylidene difluoride membrane and immunoblotted with rabbit anti-Tusc5 antibody (1 : 10000) in PBST overnight. Specific signal was detected with a horseradish peroxidase-conjugated secondary antibody (1 : 10000 goat antirabbit HRP) using Millipore Immobilon Western chemiluminescent substrate (Millipore). Blots were imaged using a Fluorochem 8800 instrument (Alpha Innotech). Densitometry intensities fell within the linear range as determined in pilot studies evaluating low to high loadings of WAT lysates.

**Statistics**

Comparisons across more than two-treatment groups in cell culture and molecular biology studies were evaluated by one-way ANOVA with a posthoc Dunnett's test comparing groups to the control (PrismGraph 4.0, GraphPad, San Diego, CA). For obese human WAT gene expression results, a repeated-measures two-way ANOVA was used to evaluate effects of obesity, WAT depot, and obesity x depot interactions: where significant interactions across variables were present, posthoc Bonferroni tests were used. Unpaired *t*-tests were used to test for significant differences between placebo- and pioglitizone-treated groups with respect to the change in WAT target gene transcript levels following treatment, or when comparing agonist-treated versus control HepG2 cells for PPAR isoform studies. For microarray studies comparing Tusc5 WAT gene expression in type 2 diabetics treated with TZDs, paired *t*-tests were employed to compare fluorescence signal values pre- versus posttreatment. Means ± SEM are presented, and *P* ≤ .05 was considered statistically significant.

## 3. Results

### 3.1. Experiments Establishing the Murine TUSC5 Gene Locus as a PPAR*γ* Target

**Figure 1:**
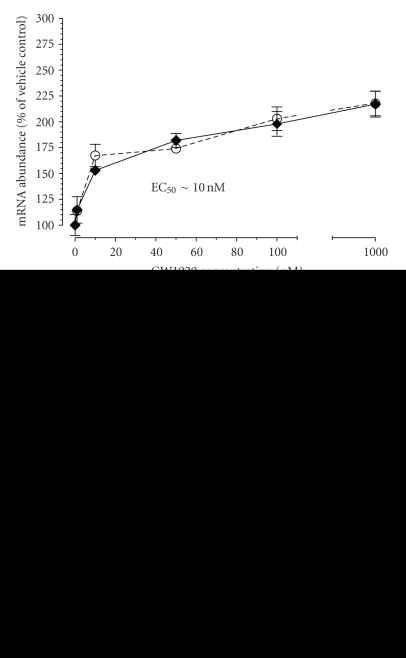
Dose-Response of Tusc5 Gene Expression to Short-term Treatment with PPAR*γ* Agonists in Mature 3T3-L1 Adipocytes. (a) Relative Tusc5 mRNA abundance in response to increasing concentrations of the potent and highly-selective non-thiazolidinedione GW1929. (b) Relative Tusc5 mRNA abundance in response to increasing concentrations of the thiazolidinedione troglitazone. Also shown for both conditions is expression of the aP2 gene, used as a positive control PPAR*γ* target gene. Symbols represent the mean ± SEM for *n* = 4/agonist concentration.

Previously, we observed in 3T3-L1 adipocytes that short-term (<24 hours) and longer-term (7 d) treatments with the non-thiazolidinedione GW1929 at a single high dose increased Tusc5 mRNA abundance and protein [2]. To ensure that this induction is via PPAR*γ* activation and not off-target events specific to GW1929, dose-response studies were conducted in mature differentiated 3T3-L1 adipocytes using two different PPAR*γ* agonists (GW1929 and troglitazone) that display large potency differences at the level of PPAR*γ* binding and activation (GW1929 is ∼35–66 times more potent; see [6]) and representing non-thiazolidinedione and TZD molecule classes, respectively. As shown in Figure 1, both agonists increased Tusc5 mRNA abundance to over 200% of that determined in control, vehicle-treated cells, with troglitazone having an approximately 50 times higher EC_50_ compared to GW1929. Also shown are mRNA level changes for the established PPAR*γ* target gene, aP2, which showed identical patterns to those seen for Tusc5. Tusc5 protein level was also increased by agonist treatment (Supplementary Figure 1in supplementary material available online at doi: 10.1155/2009/86768). The activation of Tusc5 gene expression by both agents, and the fact that the magnitude of EC_50_ differences match the known potency of these molecules for binding and activating PPAR*γ* are consistent with the concept that Tusc5 is selectively regulated by PPAR*γ* agonism.

**Figure 2:**
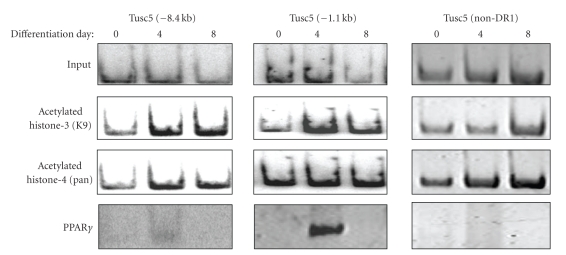
Temporal Changes in Histone Modifications and PPAR*γ* Binding at Putative DR1 Sites in the Promoter Region of the Murine TUSC5 Gene During 3T3-L1 Adipocyte Differentiation. ChIP studies were performed in preadipocytes and maturing adipocytes at days 4 and 8 days postdifferentiation initiation, employing anti-acetylated histone H3 (K9), anti-acetylated histone H4 (pan), or anti-PPAR*γ* antibodies and sequence-specific primers for putative DR1 sites (PPAR-response elements) located at promoter regions −8.4 and −1.1 kb relative to the murine TUSC5 start codon. Shown are images representative of 3 independent experiments.

A search for potential DR1 sites (PPAR*γ*-response elements) in the promoter region of murine TUSC5 revealed at least three potential sites (relative to start codon, +1) at about −1.1 kb (AGGTCATAGGCCA: −1174 to −1162), −1.8 kb (AGGTCAGAGGTTG: −1840 to −1829), and −8.4 kb (AGGTCATTGGTAA: −8467 to −8455). To determine whether PPAR*γ* protein interacts with the TUSC5 promoter in the course of normal 3T3-L1 adipocyte differentiation, a ChIP study focusing on binding at the DR1 sites was conducted, before differentiation (day 0) and at 4 and 8 d post-initiation of adipocyte differentiation. PPAR*γ* protein binding at the putative DR1 site at −1.8 kb was not detected under any condition (data not shown). Strong binding of PPAR*γ* was observed at the −1.1 kb site, but only transiently, at Day 4 (Figure 2). Observable but very modest binding could be detected at the −8.4 kb site and at Day 4 only (Figure 2). Since chromosome region-specific histone acetylation is an important factor in promoting accessibility of DNA to transcription factors, the magnitude of histone acetylation at the PPAR*γ*-binding sites was determined. There was higher histone acetylation at the putative DR1 sites of −1.1 kb and −8.4 kb with progression of adipogenesis, but this was not apparent in the non-DR1 region (Figure 2) or at the putative DR1 site at −1.8 kb that did not bind PPAR*γ* (data not shown).

**Figure 3:**
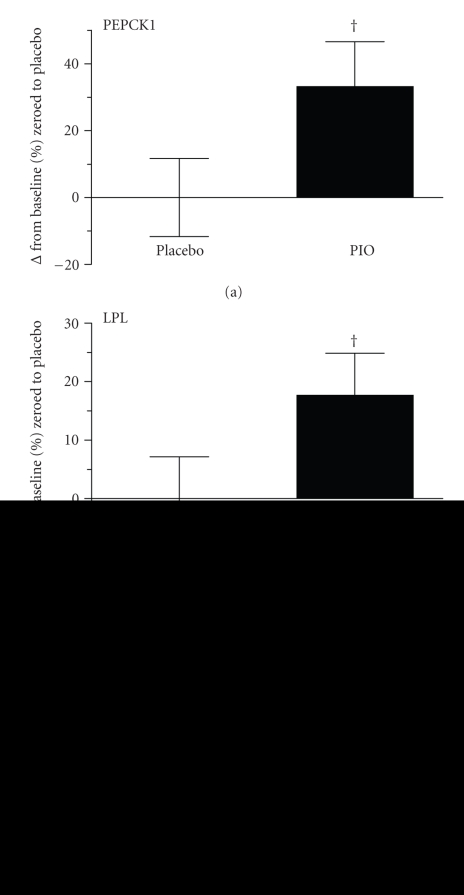
Subcutaneous WAT mRNA Expression for Tusc5 is not Increased in Type 2 Diabetic Subjects Treated >11 Weeks with Pioglitazone (PIO). Samples of subcutaneous WAT were biopsied before and after treatment with placebo (*n* = 16: 7 male, 9 female) or PIO (*n* = 16: 5 male, 11 female) and relative abundance of Tusc5 transcript was determined, using placebo group baseline expression as comparator. No change was observed in Tusc5 expression, in contrast to increases observed in mRNA levels for phosphoenolpyruvate carboxykinase 1 (PEPCK1/Pck1) and lipoprotein lipase (LPL) (^†^ *P* = .07–.09). Values are means ± SEM.

Considering the robust induction of murine adipocyte Tusc5 mRNA and protein levels by pharmacologic PPAR*γ* agonists in cell culture (Figure 1 and Supplementary Figure 1), we reasoned that if these were translatable to the clinical setting, Tusc5 transcript and protein abundance would be increased in the subcutaneous WAT of human type 2 diabetic volunteers treated with thiazolidinediones (TZDs). However, as shown in Figure 3, Tusc5 WAT transcript abundance did not increase following ≥11-week pioglitazone treatment, unlike the increases observed for the transcript levels of the established PPAR*γ* target genes PEPCK1 and LPL (*P* = .07 and *P* = .09, resp.). Notable was the large variability in Tusc5 gene expression changes over time both in the placebo- and pioglitizone-treated subjects. Using a subset of patients for whom archived WAT was available for measurement of Tusc5 protein abundance, consistent with the mRNA results there was large person-to-person variability in Tusc5 expression and no effect of pioglitazone treatment on Tusc5 protein levels when comparing pre- and post-treatment WAT (Supplementary Figure 2).

Microarray gene expression results from a separate cohort of type 2 diabetics treated with pioglitizone for 12 weeks also showed no increased Tusc5 transcript levels in the WAT when comparing pre- versus posttreatment matched samples (in arbitrary fluorescence signal units): 3084 ± 564 versus 3017 ± 601 in pre- versus posttreatment, respectively (*P* > .1). However, in subjects treated for the same period of time but using rosiglitazone, there was a trend for increased Tusc5 mRNA expression: 3132 ± 351 versus 4286 ± 433 (*P* = .07).

### 3.2. Negligible Effects of PPAR*γ* Agonism on Tusc5 Expression outside the Context of Adipocytes

The basis for the unique tissue specificity of Tusc5 expression remains unexplored, but the robust activation of Tusc5 gene expression by PPAR*γ* agonists in murine adipocytes prompted us to consider whether lack of expression in some tissues emanates from limited endogenous PPAR*γ* expression. To address this question, we examined whether manipulation of PPAR*γ* expression in liver cells could trigger Tusc5 expression.

**Figure 4:**
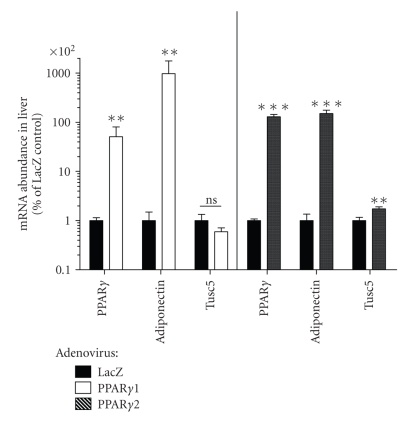
Experimental Overexpression of PPAR*γ*1 or PPAR*γ*2 in Mouse Livers in vivo Increases Adiponectin Expression but Fails to Strongly Increase Tusc5 mRNA Abundance. Mice were treated with adenoviruses containing LacZ (controls) or expression constructs to drive PPAR*γ*1 (left bars, *n* = 3/group) or PPAR*γ*2 (right bars, *n* = 6 & 7/group in controls and PPAR*γ*2, resp.). Either condition significantly increased PPAR*γ* mRNA levels and adiponectin (AdipoQ) expression relative to LacZ controls (**P < .01; ***P < .001). Effects on Tusc5 expression were either not significant (PPAR*γ*1) or modest (PPAR*γ*2; *P* < .01). Means ± SEM are depicted.

First, we opportunistically studied archived liver samples derived from mice treated with adenoviruses containing LacZ control overexpression constructs or viruses delivering PPAR*γ*1, the main PPAR*γ* in liver and most other nonadipocyte cells [12], or PPAR*γ*2 [13], the primary PPAR*γ* type in fat cells. As expected, we detected a marked induction of PPAR*γ* mRNA expression in the livers of PPAR*γ*1 or PPAR*γ*2 adenovirus-treated mice relative to controls (Figure 4, consistent with prior published reports using these samples: [12, 13]), indicating successful delivery of the transcription factors to liver in vivo. Whole liver preparations can express trace amounts of mRNA for the PPAR*γ* target gene adiponectin (AdipoQ, probably from Kuppfer cells), and following overexpression of either PPAR*γ* isoform the relative abundance of adiponectin was massively increased in relative terms (Figure 4), as expected based on prior reports using these liver samples [12, 13]. Notably, while large in relative terms, the AdipoQ induction resulted in mRNA abundance that remained <1% of that measured in a sample of murine WAT analyzed in parallel (data not shown). In contrast, trace liver Tusc5 mRNA abundance was not increased by overexpression of PPAR*γ*1 (Figure 4, left panel) and was only modestly increased to 173 ± 17% of control trace levels in PPAR*γ*2-overexpressing mice (Figure 4, right panel). Taken together, these results indicate that increased PPAR*γ*2 activity, but not PPAR*γ*1 activity, in mouse liver in vivo can induce Tusc5 gene expression, but only to levels that are a fraction of those typically determined in murine WAT and two orders of magnitude less in relative terms to the induction observed for AdipoQ.

Second, in another set of studies addressing whether Tusc5 expression can be induced in non-adipocytes overexpressing PPAR*γ*, human HepG2 hepatoblastoma stable cell lines containing Tet-off constructs (HepG2-tet-off-hPPAR cells: PPAR isoforms *α*, *β*/*δ*, *γ*1, *γ*2) were examined under conditions in which PPAR expression was induced, with or without treatment with specific ligands to trigger the PPAR isoform activities. The conditions mimicked those described previously that demonstrated successful induction of PPAR target genes in this model [14]. Unlike positive control human WAT cDNA analyzed in parallel, Tusc5 mRNA was nondetectable in HepG2 cells under all conditions (transcripts for leptin and AdipoQ/adiponectin were also not detected). The lack of Tusc5 induction in HepG2 cells contrasted with a variety of PPAR target genes that were induced under conditions of PPAR activation (Table 1). Of these, PEPCK1 and ADFP were induced strongly by agonist treatment in all PPAR isoform-expressing cells. ACOX1 and CPT1b were induced by PPAR*α* agonism, but interestingly ACOX1 was also significantly increased by PPAR*δ* and PPAR*γ* agonists in cells expressing those transcription factors. CPT1b transcript levels, in contrast, were not increased by PPAR*γ* activation.

### 3.3. Lack of Association of Tusc5 Coding Sequence Polymorphisms and WAT Gene Expression in Human Obesity

**Effects of Human Obesity and WAT Depot Site on Tusc5 mRNA Expression**

**Figure 5:**
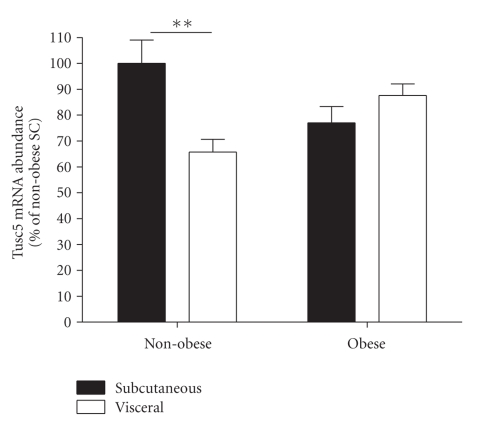
Adipose Tusc5 Transcript Levels are not Significantly Altered in Obese Adult Women. Samples from a cohort of obese (*n* = 12) and non-obese (*n* = 10) women [15] were used to assay mRNA abundance for Tusc5 by quantitative RT-PCR in subcutaneous (SC) and visceral (omental) WAT. Means ± SEM are depicted, with non-obese SC mRNA levels considered 100%. There were no significant effects of obesity, but depot (*P* = .05) and depot x obesity interaction (*P* < .0001) were observed due to the significantly lower Tusc5 expression in the visceral versus subcutaneous WAT of nonobese volunteers (**P < .01).

Matched subcutaneous (SC) and visceral (omental) adipose tissue mRNA samples from a cohort of obese and nonobese French women, previously described in an expression profile study of adipose 11ß-hydroxysteroid dehydrogenase [15] and synuclein-*γ* [16], were used to determine whether Tusc5 is differentially-expressed in human obesity or across fat depots. Relative to non-obese SC WAT, Tusc5 mRNA abundance was ∼20% reduced in obese SC and visceral WAT (Figure 5), but this modest effect of obesity was not statistically significant. A significant depot effect (*P* = .05) and depot × obesity interaction (*P* < .001) was observed, explained by the significantly lower Tusc5 expression in the visceral versus SC WAT of non-obese subjects that was not observed in obese persons (Figure 5).

**TUSC5 Coding Variants in Obese and Lean Humans**

**Figure 6:**
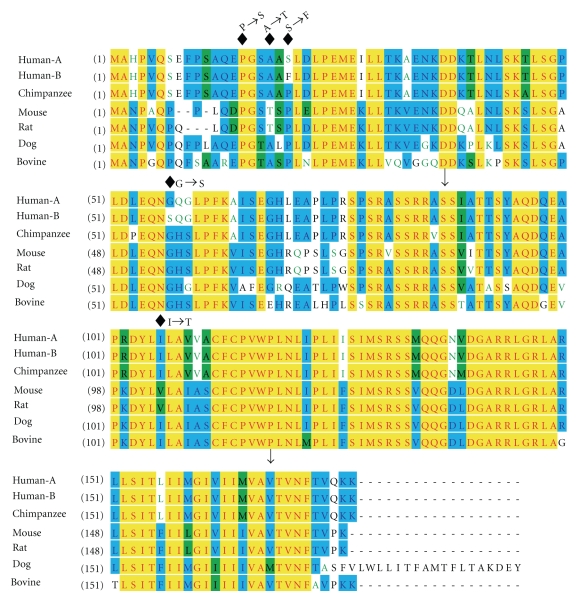
Locations of Single-Nucleotide Polymorphisms (SNPs) Impacting the Coding Region of Human TUSC5. A number of SNPs were detected in the course of resequencing the TUSC5 open reading frame of >700 human subjects (see Table 2), with 5 SNPs encoding amino acid changes (see diamonds; residue changes are noted). Notably, the I106T shift is within the CD225 domain of Tusc5 (domain bordered by arrows), and the P15S change is at a site highly-conserved across species. The alignment tool in VectorNTI (Invitrogen) was used to compare known and putative Tusc5 GenBank sequences from two human entries (BAC43751 [HUMAN-A] and NP_758955 [HUMAN-B]), chimpanzees (XP_001153397), mice (NP_808377), rats (NP_001034252), dogs (XP_548306), and cattle (XP_605998).

We sequenced TUSC5 exons and intron/exon boundaries in very lean and very obese subjects to determine whether rare or common coding variants are associated with differences in human adiposity. Five missense and two sense mutations were identified, but the genotype frequencies of each did not differ between lean and obese persons, and no nonsense or frameshift mutations were detected (Table 2). Two rare variants predict conserved amino acid sequences (V109V & A167A). One rare missense mutation (A18T) that results in substitution of uncharged, polar threonine for nonpolar hydrophobic alanine had allele frequencies of 0.017 and 0.016 in obese and lean persons, respectively. Several other relatively common missense mutations were observed: proline to uncharged polar serine (P15S), serine to nonpolar hydrophobic phenylalanine (S20F), and uncharged polar glycine to serine (G57S). Finally, 6 lean and 3 obese subjects were heterozygous carriers of a rare missense mutation (I106T) that would result in a switch from nonpolar hydrophobic isoleucine to threonine, and this sequence variation is found in the highly conserved CD225 domain of Tusc5 (see Figure 6). The amino acid changes associated with these missense mutations are highlighted in a mammalian Tusc5 alignment (Figure 6) and notably the S20F and G57S variants identified above were also observed in the human Tusc5 GenBank protein sequence entries.

## 4. Discussion

Tusc5 displays an interesting biology, in that it is robustly coexpressed in both adipocytes and peripheral afferent neurons, but its function has remained elusive and very little is known about the molecular regulation of Tusc5 gene expression. Findings to date indicate that cold ambient temperature markedly reduces rodent WAT and BAT Tusc5 mRNA levels, and Tusc5 expression rises during adipogenesis in white and brown adipocytes [2, 4, 5]. The cold-suppression of Tusc5 expression does not appear to be via activation of *β*3-adrenergic receptors [5]. Up regulation of Tusc5 gene expression by a single concentration of the PPAR*γ* agonist GW1929 in immature and mature 3T3-L1 adipocytes [2] led us to assess whether murine TUSC5 gene is a bona fide PPAR*γ* target gene. PPAR*γ* targets specifically or highly expressed in adipocytes are typically implicated in pathways important to metabolic regulation, so characterizing the biology of Tusc5 and its association with PPAR*γ* should further illuminate its role in metabolism and adipose tissue function.

Results herein confirm that the murine TUSC5 gene is a PPAR*γ* target in adipocytes and highlight that the interrelationship between the TUSC5 gene and PPAR*γ* is quite complex. First, dose-response experiments in 3T3-L1 adipocytes using a TZD PPAR*γ* agonist (troglitazone) and a non-TZD agonist (GW1929) resulted in significantly increased Tusc5 mRNA abundance, with EC_50_ differences that matched the known potencies of these compounds for the PPAR*γ* receptor (see [6]). This suggests that up-regulation of Tusc5 expression by these compounds is not due to off-target effects and is not agonist class specific. Second, chromatin immunoprecipitation studies proved that endogenous murine PPAR*γ* directly binds one predicted TUSC5 DR1 site strongly (a −1.1 kb PPAR-response element) and another weakly (−8.4 kb site) during 3T3-L1 adipogenesis. While these results establish that interactions take place between PPAR*γ* and the murine TUSC5 gene in 3T3-L1 adipocytes, PPAR*γ* binding to murine TUSC5 promoter elements was transient (Figure 2) and only detected at a mid-stage of adipocyte maturation (Day 4 post-differentiation) but not in more mature fat cells (Day 8). The basis for this is not known, but time-dependent shifts in concentrations of endogenous ligands and/or changes in activators/repressors of PPAR*γ* DNA binding are likely explanations. There is precedent for the latter. Activity of PPAR*γ* at the LPL gene promoter is attenuated upon complex formation of ligand-bound PPAR*γ* to hypophosphorylated retinoblastoma protein (RB) and histone deacetylase 3 (HDAC3) [18]. This inhibitory complex is adipogenesis stage-dependent, such that earlier in adipogenesis (i.e., at Day 4), hyperphosphorylation of RB minimizes complex formation (thus enabling maximal PPAR*γ* activity at the LPL promoter), but later in adipogenesis (at Day 8) PPAR*γ*-RB-HDAC3 complex formation is more apparent [18]. Nevertheless, expression of LPL remains robust in fully developed adipocytes [2, 18, 19], indicating that other factors sustain LPL expression in the face of reduced PPAR*γ* binding to the LPL promoter later in adipogenesis. It remains to be established in future studies whether the PPAR*γ*-RB-HDAC3 interaction plays a role in temporal regulation of the TUSC5 gene locus in maturing fat cells.

There is some support for the idea that additional DR1 sites participate in driving net Tusc5 gene expression. Coupling ChIP with whole genome tiling arrays (ChIP-chip approach) in fully mature, 10 day postdifferentiated 3T3-L1 adipocytes, Lazar and colleagues identified three 3′ UTR PPAR*γ* binding sites on the murine TUSC5 gene (see PPAR_4579, PPAR_4580, and PPAR_4581 of Supplementary Table 1 in [20]). These investigators did not observe PPAR*γ* binding to the −1.1 kb DR1 site, not unexpected considering our results showing that binding drops off after 4 days of adipogenesis. An additional site within the first intron of TUSC5 was also observed upon further examination of the raw ChIP-chip data (Lefterova and Lazar, personal communication), and intronic PPAR*γ* associations can clearly influence gene expression (i.e., for retinol saturase, [21]). Thus, additional research employing a variety of TUSC5 promoter-intronic-luciferase constructs with or without PPAR*γ* ligand treatment will be required to help unravel how these PPAR*γ* binding sites in isolation or in combination alter gene expression.

The complicated relationship between PPAR*γ* and Tusc5 was further highlighted by opportunistic analysis of archived WAT samples from clinical studies of type 2 diabetics treated for >11 weeks with the PPAR*γ* agonist pioglitazone [8], in which we did not detect an increase in WAT Tusc5 mRNA by quantitative PCR or protein despite increases in expression of other target genes (Figure 3). Microarray adipose tissue expression data from a different human TZD study in type 2 diabetics confirmed the observation above that pioglitazone treatment does not increase Tusc5 mRNA levels. In contrast, rosiglitazone tended to increase Tusc5 mRNA levels, but this was highly variable among the individuals tested. This latter observation may be due to ligand-selective gene regulation (i.e., [22]) that is explained by the selective PPAR modulator (SPPARM) model [23]. Under this model, ligand-bound PPAR*γ* molecules can assume ligand-specific 3-dimensional structures that confer ligand-specific transcriptional activity through differential cofactor interaction and DNA binding-specificity. The lack of effect of PIO in triggering WAT Tusc5 expression in our human cohorts (in contrast to the robust increase in murine adipocytes) might point to potential species-specific differences in the Tusc5-PPAR*γ* association, or large variability in this association across individuals differing in metabolic status or disease severity. Finally, considering that samples were obtained only at a single late timepoint following pioglitazone treatment, it is possible that WAT Tusc5 expression was actively regulated during the more dynamic phase of early TZD treatment.

Several PPAR*γ* target genes, including the adipocyte marker adiponectin, can be induced in non-adipocytes by experimental overexpression of PPAR*γ*, indicating that the latter is sufficient for activation of “adipocyte-specific” gene expression outside the context of fat tissue. For example, adenoviral delivery of PPAR*γ*1 in mouse liver markedly increased adiponectin mRNA levels and increased liver fat accumulation [12]. Similarly, adenoviral injection of PPAR*γ*2 in mice resulted in a lipogenic liver phenotype and induction of several PPAR*γ* target genes [13]. We reasoned that Tusc5 expression, like that of the classic PPAR*γ* target adiponectin, should be up-regulated by expressing PPAR*γ* isoforms in liver. However, liver Tusc5 mRNA changes following hepatic PPAR*γ* overexpression in vivo were nominal at best, especially when compared to a massive relative increase in adiponectin mRNA levels. Expression of Tusc5 could not be induced in the human hepatocyte cell line HepG2 even after maneuvers that increased PPAR activity and hence expression of assorted PPAR target genes (Table 2; also see [14]). These results indicate that in contrast to adiponectin and some other PPAR*γ* target genes, factors present in adipocytes but lacking in liver are required for induction of the Tusc5 gene by endogenous or pharmacologic PPAR*γ* agonists. A non-adipocyte cell type that strongly expresses Tusc5 is the peripheral afferent neuron, so it will be interesting to determine if Tusc5 expression can be increased by PPAR*γ* agonists in these cells.

Similar to the pattern observed for other genes that mark the adipocyte maturation process (adiponectin, ADD1, aP2, e.g.) [24], we have demonstrated that early in adipogenesis histone acetylation at Tusc5 PPAR*γ* binding sites increases. Such site-specific chromosomal modifications are associated with access for gene regulatory factors including PPAR*γ*, best exemplified by cyclin D1 inhibition of PPAR*γ* binding to LPL and aP2 DR1 sites via HDAC recruitment and reduced histone H3 acetylation [25, 26]. Increased histone acetylation in adipocyte genes is due in part to marked down-regulation of histone deacetylase (HDAC) enzyme levels and reduced interaction of HDACs with mature adipocyte marker gene loci several days following induction of 3T3-L1 adipocyte differentiation [24]. It is notable that the transition from limited Tusc5 gene histone acetylation in preadipocytes to increased acetylation by at least 4 days following the start of adipocyte differentiation is consistent with the induction kinetics of Tusc5 gene expression in this cell model [2]. Therefore, these results are consistent with the hypothesis that Tusc5 expression, like other genes triggered during the adipogenesis program, is regulated through increased histone acetylation at PPAR*γ* chromosomal binding sites within the murine Tusc5 gene. Interestingly, PPAR*γ* binding to the Tusc5 promoter was transient despite maintenance of histone acetylation at the DR1 sites throughout 3T3-L1 adipocyte maturation, suggesting that acetylation and hence concomitant transcription factor accessibility to these sites does not alone explain temporal changes in PPAR*γ* binding.

Despite emerging evidence for a role for Tusc5 in adipocyte function, robust WAT expression of the gene, and its regulation by PPAR*γ* agonists in cultured adipocytes, it is not known if Tusc5 expression or activity is associated with obesity phenotypes. Only one other study has addressed this question, using Zucker fatty rats that lack proper leptin signaling and therefore become extremely obese: Tusc5 mRNA and protein levels were very slightly increased in subcutaneous WAT, unchanged in mesenteric, and yet decreased substantially in epididymal WAT [4]. We observed no significant difference between adult obese and non-obese women in terms of WAT Tusc5 transcript levels (Figure 5). The frequencies of TUSC5 coding sequence variants in 758 lean and obese volunteers determined if rare or common variants are associated with body composition phenotypes in the human population. Several SNPs were identified, but the frequencies of these were not different when comparing lean and obese subjects (see Table 2). Mutations leading to altered amino acid residues conserved across mammalian Tusc5 orthologues (or in the CD225 domain) are predicted to lead to physiologically-important protein functional changes, and notably an I106T shift in a highly-conserved portion of the CD225 domain was detected and introduces a charged residue at that site (Figure 6). The functional implications for this remain to be evaluated. Thus, our results do not support the hypothesis that WAT gene expression differences or coding polymorphisms in TUSC5 are associated with lean or obese phenotypes.

In conclusion, these experiments have established that the murine TUSC5 gene is a PPAR*γ*-regulated locus in adipocytes, but whether this association occurs in human fat cells remains to be established. The PPAR*γ*-TUSC5 interaction is highly-complex, temporally-regulated during adipogenesis, and involves tissue specific factors driving expression of the gene in adipocytes in response to experimental PPAR*γ* agonism. There is little evidence for a major role for Tusc5 WAT expression differences in obesity development, and although we identified several missense mutations in the TUSC5 gene locus, none was associated with adiposity in a large human cohort. Therefore, it appears that future studies of Tusc5 biology should focus less on the association of the protein with adipose tissue abundance phenotypes and more on its participation in other facets of adipocyte function.

## Supplementary Material

Tusc5 protein abundances were measured in 3T3-L1 adipocytes or human subcutaneous white adipose tissue (WAT) after PPAR*γ* agonist treatment. Supplementary Figure 1: Treatment of Mature Murine 3T3-L1 Adipocytes with the PPAR*γ* Agonist GW1929 or Troglitazone (Trog) Increases Tusc5 Protein Abundance. Cells treated in parallel with those used for dose-response studies depicted in Figure 1 of the manuscript were used to isolate protein and perform Western blot analysis (see Methods). Both GW1929 and Trog increased Tusc5 protein expression. Blot depicts results from *n* = 2 samples/treatment and is representative of the experiment. Supplementary Figure 2: Tusc5 Protein Expression in Subcutaneous WAT is Unchanged by >11 Wk of Pioglitazone (PIO) Treatment in Type 2 Diabetic Adults. WAT samples available from a subset of subjects for whom gene expression analyses were performed (see Figure 3 in the manuscript) were used for Western blot determination of Tsuc5 protein abundance before and after treatment with placebo or PIO. Wide person-to-person variability in Tusc5 protein levels was observed, and no effect of PIO treatment was apparent, consistent with mRNA patterns (see Figure 3 in manuscript).

## Figures and Tables

**Table 1:** Relative transcript abundances of PPAR target genes in HepG2 cells experimentally-expressing different PPAR isoforms, in the presence or absence of isoform-selective agonists.

PPAR isoform expressed^‡^	PPAR agonist treatment^†^		
	−	+	Treatment effect
			*P < .05
			**P < .01
			***P < .001
PPAR*α*			
Tusc5, adiponectin (adipoQ), leptin (ob)	n.d.	n.d.	
PEPCK1 (Pck1)	100 ± 7%	401 ± 54%	∗∗
ACOX1	100 ± 5%	177 ± 4%	∗∗∗
CPT1b	100 ± 6%	154 ± 14%	∗
ADFP	100 ± 13%	560 ± 41%	∗∗∗

PPAR*δ*			
Tusc5, adiponectin (adipoQ), leptin (ob)	n.d.	n.d.	
PEPCK1 (Pck1)	100 ± 16%	548 ± 90%	∗∗
ACOX1	100 ± 3%	236 ± 13%	∗∗∗
CPT1b	100 ± 4%	137 ± 8%	∗
ADFP	100 ± 4%	1830 ± 153%	∗∗∗

PPAR*γ*1			
Tusc5, adiponectin (adipoQ), leptin (ob)	n.d.	n.d.	
PEPCK1 (Pck1)	100 ± 5%	336 ± 31%	∗∗
ACOX1	100 ± 7%	162 ± 1%	∗∗
CPT1b	100 ± 1%	114 ± 12%	
ADFP	100 ± 11%	281 ± 11%	∗∗∗

PPAR*γ*2			
Tusc5, adiponectin (adipoQ), leptin (ob)	n.d.	n.d.	
PEPCK1 (Pck1)	100 ± 3%	534 ± 113%	∗
ACOX1	100 ± 3%	172 ± 22%	∗
CPT1b	100 ± 9%	88 ± 2%	
ADFP	100 ± 19%	278 ± 64%	*P* = .056

^‡^Using HepG2 Tet-off human PPAR isoform-specific overexpressing stable cell lines [14] (see Methods); *n* = 3/treatment.

^†^PPAR isoform-selective agonists used for PPAR*α*, *δ*, *γ*1, *γ*2 experiments were, respectively, GW7647 (1 *μ*M), GW501516 (100 nM), and rosiglitazone (1 *μ*M); cells were treated for 24 hours prior to mRNA collection.

n.d.: not detectable (note that Tusc5, adiponectin, and leptin transcripts were readily detected in a human WAT sample run in parallel).

**Table 2:** Frequencies of rare and common coding variants in TUSC5 in lean and obese subjects.

Amino acid change (nucleotide shift)	Allele frequency	
	Lean	Obese
P15S (C→T)	CC = 317	CC = 318
	CT = 58	CT = 61
	TT = 2	TT = 2

A18T(G→A)	GG = 365	GG = 368
	AG = 12	AG = 12
	AA = 0	AA = 1

S20F(C→T)	CC = 180	CC = 197
	CT = 172	CT = 151
	TT = 25	TT = 33

G57S(G→A)	GG = 210	GG = 217
	AG = 146	AG = 139
	AA = 21	AA = 25

I106T(T→C)	TT = 371	TT = 378
	CT = 6	CT = 3
	CC = 0	CC = 0

V109V(C→T)	CC = 376	CC = 381
	CT = 1	CT = 0
	TT = 0	TT = 0

A167A(C→T)	CC = 160	CC = 375
	CT = 0	CT = 1
	TT = 0	TT = 0

## References

[1] Konishi H, Sugiyama M, Mizuno K (2003). Detailed characterization of a homozygously deleted region corresponding to a candidate tumor suppressor locus at distal 17p13.3 in human lung cancer. *Oncogene*.

[2] Oort PJ, Warden CH, Baumann TK, Knotts TA, Adams SH (2007). Characterization of Tusc5, an adipocyte gene co-expressed in peripheral neurons. *Molecular and Cellular Endocrinology*.

[3] Deblandre GA, Marinx OP, Evans SS (1995). Expression cloning of an interferon-inducible 17-kDa membrane protein implicated in the control of cell growth. *The Journal of Biological Chemistry*.

[4] Shibata T, Koide H, Hayashi R (2007). Molecular cloning and characterization of rat brain endothelial cell derived gene-1 (tumor suppressor candidate 5) expressing abundantly in adipose tissues. *Molecular and Cellular Endocrinology*.

[5] Koide H, Shibata T, Yamada N (2007). Tumor suppressor candidate 5 (TUSC5) is expressed in brown adipocytes. *Biochemical and Biophysical Research Communications*.

[6] Brown KK, Henke BR, Blanchard SG (1999). A novel N-aryl tyrosine activator of peroxisome proliferator-activated receptor-*γ* reverses the diabetic phenotype of the Zucker diabetic fatty rat. *Diabetes*.

[7] Seo JB, Noh MJ, Yoo EJ (2003). Functional characterization of the human resistin promoter with adipocyte determination- and differentiation-dependent factor 1/sterol regulatory element binding protein 1c and CCAAT enhancer binding protein-*α*. *Molecular Endocrinology*.

[8] Bogacka I, Xie H, Bray GA, Smith SR (2004). The effect of pioglitazone on peroxisome proliferator-activated receptor-*γ* target genes related to lipid storage in vivo. *Diabetes Care*.

[9] Bandyopadhyay GK, Yu JG, Ofrecio J, Olefsky JM (2006). Increased malonyl-CoA levels in muscle from obese and type 2 diabetic subjects lead to decreased fatty acid oxidation and increased lipogenesis; thiazolidinedione treatment reverses these defects. *Diabetes*.

[10] Yu JG, Javorschi S, Hevener AL (2002). The effect of thiazolidinediones on plasma adiponectin levels in normal, obese, and type 2 diabetic subjects. *Diabetes*.

[11] Sears DD, Hsiao G, Hsiao A (2009). Mechanisms of human insulin resistance and thiazolidinedione-mediated insulin sensitization. *Proceedings of the National Academy of Sciences of the United States of America*.

[12] Yu S, Matsusue K, Kashireddy P (2003). Adipocyte-specific gene expression and adipogenic steatosis in the mouse liver due to peroxisome proliferator-activated receptor *γ*1 (PPAR*γ*1) overexpression. *The Journal of Biological Chemistry*.

[13] Uno K, Katagiri H, Yamada T (2006). Neuronal pathway from the liver modulates energy expenditure and systemic insulin sensitivity. *Science*.

[14] Tachibana K, Kobayashi Y, Tanaka T (2005). Gene expression profiling of potential peroxisome proliferator-activated receptor (PPAR) target genes in human hepatoblastoma cell lines inducibly expressing different PPAR isoforms. *Nuclear Receptor*.

[15] Desbriere R, Vuaroqueaux V, Achard V (2006). 11*β*-hydroxysteroid dehydrogenase type 1 mRNA is increased in both visceral and subcutaneous adipose tissue of obese patients. *Obesity*.

[16] Oort PJ, Knotts TA, Grino M (2008). *γ*-synuclein is an adipocyte-neuron gene coordinately expressed with leptin and increased in human obesity. *Journal of Nutrition*.

[17] Ahituv N, Kavaslar N, Schackwitz W (2006). A *PYY* Q62P variant linked to human obesity. *Human Molecular Genetics*.

[18] Fajas L, Egler V, Reiter R (2002). The retinoblastoma-histone deacetylase 3 complex inhibits PPAR*γ* and adipocyte differentiation. *Developmental Cell*.

[19] Morrison RF, Farmer SR (1999). Role of PPARgamma in regulating a cascade expression of cyclin-dependent kinase inhibitors, p18(INK4c) and p21(Waf1/Cip1), during adipogenesis. *The Journal of Biological Chemistry*.

[20] Lefterova MI, Zhang Y, Steger DJ (2008). PPAR*γ* and C/EBP factors orchestrate adipocyte biology via adjacent binding on a genome-wide scale. *Genes and Development*.

[21] Schupp M, Lefterova MI, Janke J (2009). Retinol saturase promotes adipogenesis and is downregulated in obesity. *Proceedings of the National Academy of Sciences of the United States of America*.

[22] Sears DD, Hsiao A, Ofrecio JM, Chapman J, He W, Olefsky JM (2007). Selective modulation of promoter recruitment and transcriptional activity of PPAR*γ*. *Biochemical and Biophysical Research Communications*.

[23] Olefsky JM (2000). Treatment of insulin resistance with peroxisome proliferator-activated receptor gamma agonists. *The Journal of Clinical Investigation*.

[24] Yoo EJ, Chung J-J, Choe SS, Kim KH, Kim JB (2006). Down-regulation of histone deacetylases stimulates adipocyte differentiation. *The Journal of Biological Chemistry*.

[25] Fu M, Rao M, Bouras T (2005). Cyclin D1 inhibits peroxisome proliferator-activated receptor *γ*-mediated adipogenesis through histone deacetylase recruitment. *The Journal of Biological Chemistry*.

[26] Fu M, Wang C, Rao M (2005). Cyclin D1 represses p300 transactivation through a cyclin-dependent kinase-independent mechanism. *The Journal of Biological Chemistry*.

