# Supplementary figures and images for: Molecular Characterization of the Tumor Suppressor Candidate 5 Gene: Regulation by PPARγ and Identification of TUSC5 Coding Variants in Lean and Obese Humans
Source: PPAR Res. 2010 Mar 1;2009:867678. doi: 10.1155/2009/867678 (PMC2830574; doi:10.1155/2009/867678)

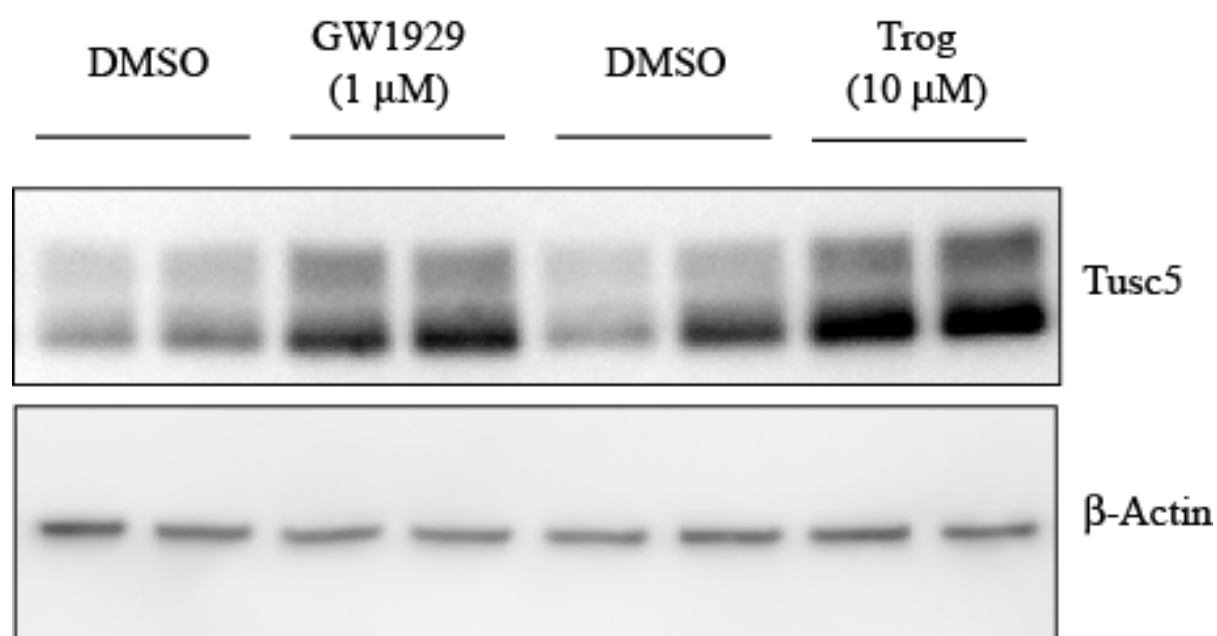

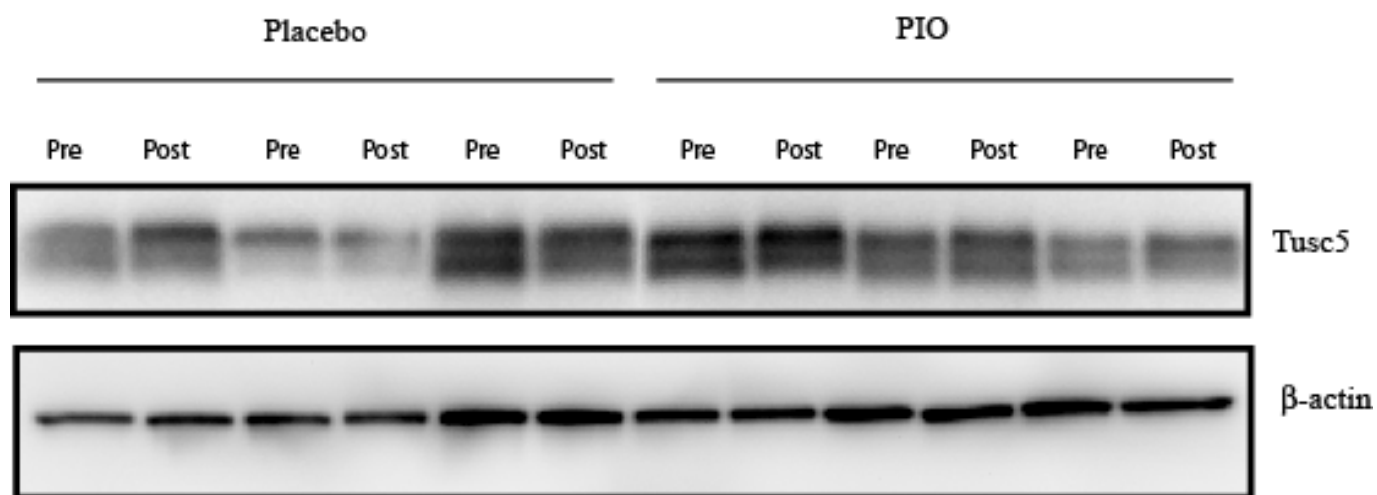

Supplement: Supplementary file 1 — Tusc5 protein abundances were measured in 3T3-L1 adipocytes or human subcutaneous white adipose tissue (WAT) after PPARγ agonist treatment. Supplementary Figure 1: Treatment of Mature Murine 3T3-L1 Adipocytes with the PPARγ Agonist GW1929 or Troglitazone (Trog) Increases Tusc5 Protein Abundance. Cells treated in parallel with those used for dose-response studies depicted in Figure 1 of the manuscript were used to isolate protein and perform Western blot analysis (see Methods). Both GW1929 and Trog increased Tusc5 protein expression. Blot depicts results from n = 2 samples/treatment and is representative of the experiment. Supplementary Figure 2: Tusc5 Protein Expression in Subcutaneous WAT is Unchanged by >11 Wk of Pioglitazone (PIO) Treatment in Type 2 Diabetic Adults. WAT samples available from a subset of subjects for whom gene expression analyses were performed (see Figure 3 in the manuscript) were used for Western blot determination of Tsuc5 protein abundance before and after treatment with placebo or PIO. Wide person-to-person variability in Tusc5 protein levels was observed, and no effect of PIO treatment was apparent, consistent with mRNA patterns (see Figure 3 in manuscript). [file 867678.f1.pdf]
